# Suspect Screening for PFAS in Groundwater with an Accessible LC–MS Workflow

**DOI:** 10.1021/acsomega.5c08713

**Published:** 2026-03-20

**Authors:** Bianca F. da Silva, Kya N. Bruckner, Sonia C. N. Queiroz, Carla B. G. Bottoli, Jon Chorover, Leif Abrell

**Affiliations:** † Institute of Chemistry, National Institute of Science and Technology of Bioanalytics Lauro Kubota, 28132 Universidade Estadual de Campinas, Cidade Universitária Zeferino Vaz, 13083-970 Campinas, SP, Brazil; ‡ Department of Environmental Science, 8041 The University of Arizona, 85721 Tucson, AZ, United States; § Laboratório de Resíduos e Contaminantes, Embrapa Meio Ambiente, Rodovia SP 340, km 127.5, 13918-110 Jaguariúna, SP, Brazil

## Abstract

Groundwater in North America is contaminated with per- and polyfluoroalkyl substances (PFAS) at more than 9500 locations. A major source of this contamination is aqueous film-forming foam (AFFF), widely used for fire suppression at military facilities and airfields. Many of the thousands of PFAS remain poorly characterized and are not amenable to targeted quantitative analytical methods, which allow them to remain undetected. Suspect screening, an analytical strategy that searches for potential or likely compounds from a predefined list without requiring analytical reference standards, combined with liquid chromatography-high resolution tandem mass spectrometry (LC-HRMS/MS), has emerged as an alternative or complementary approach to classical targeted analysis. Herein, an accessible suspect screening workflow was developed using data-dependent acquisitions and the NIST suspect list of 4712 PFAS, processed with TraceFinder software, followed by FreeStyle MS^2^ spectral management. Eleven PFAS were identified in AFFF-impacted groundwaters, including six compounds previously undetected by targeted experiments: 1*H*-perfluoropentane, 1*H*-perfluoroheptane, perfluorobutylsulfonamide (FBSA), perfluorohexanesulfonamide (FHxSA), perfluoropropanesulfonamide (FPrSA), and perfluoropropanesulfonic acid (PFPrS). Direct sample injection imposed sensitivity limitations, likely preventing the detection of additional PFAS present at lower concentrations. Nevertheless, the simplicity and reduced software investment requirements of this workflow make it a promising approach for broad adoption by the scientific community.

## Graphical abstract

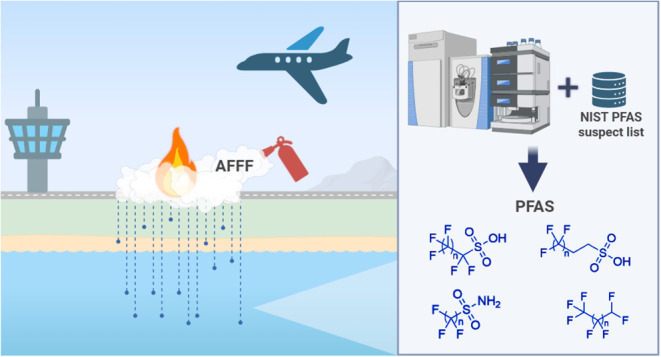

## 1 Introduction

More than 9500 locations in North America have groundwater contaminated with perfluoroalkyl and polyfluoroalkyl substances (PFAS).^1^ Aqueous film-forming foams (AFFF), widely deployed for fire suppression at military facilities and airfields, represent a major source of this contamination in groundwater.^2^ The exceptional stability of C–F bonds enables PFAS to function effectively under extreme conditions, and their high persistence has led to their classification as “forever chemicals”.^3^ These properties, thermal resistance, chemical inertness, and repellency to water and oils, have supported extensive commercial and industrial use, including in nonstick cookware, stain- and water-repellent textiles, waterproofing agents, and food packaging. An enormous variety of PFAS structural types is recognized, with estimates suggesting that there are over 4700 types of PFAS,^4^ and other references list over 12,000 substances.^5^ As a result, thousands of PFAS occur in the environment.^4^

Concern regarding PFAS toxicity has grown substantially. Exposure has been associated with carcinogenic outcomes, immune and endocrine disruption, neurotoxicity, reproductive and developmental effects,^3,6^ and bioaccumulation with biomagnification across food webs.^3,4^ Regulatory actions reflect these concerns, for example, with respect to drinking water: the U.S. Environmental Protection Agency (EPA), which added perfluorooctanoic acid (PFOA) and perfluorooctanesulfonic acid (PFOS) to the Contaminant Candidate List (CCL) in 2009, and in 2024, established maximum contaminant levels (MCLs) for PFOA, PFOS, perfluorohexanesulfonic acid (PFHxS), perfluorononanoic acid (PFNA), hexafluoropropylene oxide dimer acid (HFPO-DA), and perfluorobutanesulfonic acid (PFBS, in mixtures).^7^ On a broader scale, PFAS are now considered part of the “novel entities” planetary boundary, one of nine biophysical limits proposed to define a safe operating space for humanity, a boundary related to chemical pollution that has already been exceeded.^8,9^

Despite the environmental ubiquity and regulatory significance of PFAS, the analytical coverage remains limited. Among the thousands of PFAS known, only a small subset can be quantified using validated targeted methods.^10^ For example, EPA Method 1633A, widely applied for groundwater monitoring, quantifies just 40 PFAS using liquid chromatography-tandem mass spectrometry (LC–MS/MS) with multiple reaction monitoring (MRM).^11^ Such targeted methods (including EPA Methods 533 and 537.1) depend on authentic standards and validated precursor/product ion transitions, resources that do not exist for the vast majority of PFAS.^12,13^ Consequently, many PFAS remain analytically “hidden”.^10^

High resolution tandem mass spectrometry (HRMS/MS), including quadrupole-Orbitrap instruments, enables broader chemical coverage by providing accurate mass, isotopic patterns, and informative MS^2^ (product ions) spectra.^14^ LC-HRMS/MS-based suspect screening has therefore emerged as a robust alternative or complement to traditional targeted analysis.^15,16^ Suspect screening relies on prior information (e.g., structural libraries or suspect lists) to identify potential PFAS without the requirement of analytical standards. While both suspect screening and nontarget screening employ similar HRMS data acquisitions, they differ substantially in analytical complexity.^17^ Suspect screening is generally more feasible than nontarget workflows when considering time, cost, productivity,^18^ and accessibility: it offers higher annotation rates, reduced computational demands, and greater practicality for routine laboratories.

However, many laboratories, particularly those focused on environmental monitoring, lack the specialized, proprietary, or advanced computational tools required for sophisticated nontarget data analysis. As a result, a critical analytical gap has emerged: targeted LC–MS/MS methods are accessible but detect only a small subset of PFAS, whereas comprehensive nontarget workflows detect many more PFAS but are difficult to implement due to resource and expertise constraints. Bridging this gap requires accessible HRMS-based strategies that expand PFAS detection, while minimizing technical and financial barriers.

With this need in mind, we developed a suspect screening workflow that provides expanded PFAS coverage without the requirement of advanced software or custom computational pipelines. This approach uses data-dependent acquisition (DDA), with screening guided by a freely available suspect list of 4712 PFAS from the National Institute of Standards and Technology (NIST) and leverages readily accessible software tools. We demonstrate the utility of this workflow by identifying six nontarget PFAS without analytical standards (confidence Level 2), including 1*H*-perfluoroalkanes and sulfonamides. The attractiveness of LC-HRMS/MS-based PFAS identification continues to grow, with applications in AFFF-impacted sites,^19^ surface waters,^20^ groundwater,^21,22^ soils,^22^ biota such as fish^23^ and marine mammals,^24^ and assessments of human exposure, including drinking water^25^ and firefighters’ blood.^26^ The relatively low-cost software tools used in the workflow described here may enable broader implementation of suspect screening across environmental laboratories.

In this study, we applied the proposed accessible workflow using high performance liquid chromatography (HPLC) coupled to a quadrupole-Orbitrap HRMS/MS system. Groundwater samples were collected from aquifers in Arizona impacted by historical AFFF use, specifically the Central Tucson Basin near Davis-Monthan Air Force Base and the Western Salt River Valley near Luke Air Force Base, along with two wells from Willow Grove, Pennsylvania, a historically contaminated site.^10^ AFFF formulations contain as many as 60 PFAS classes and represent a rich point source of PFAS chemical diversity in contaminated aquifers, much of which remains undetectable using targeted LC–MS/MS.^27,28^ Samples were analyzed by direct injection, intentionally avoiding extraction and concentration steps to evaluate intrinsic sensitivity and workflow performance without confounding matrix-recovery variability. Consequently, PFAS present at lower concentrations would be missed if their precursor ions are not selected for fragmentation in DDA due to insufficient abundance.

These sample locations also have comparative data from previous studies, including total oxidizable precursor (TOP) assays, and total organic fluorine (TOF) quantification via combustion ion chromatography (CIC),^10^ techniques that capture nontarget PFAS but provide limited structural information. Collectively, these data sets provide a complementary framework for evaluating the performance and added value of the accessible suspect screening workflow presented here.

## 2 Materials and Methods

### 2.1 Sample Collection

Five raw groundwater samples were collected from, or nearby, military airfields with historic AFFF use. Samples 1 and 2 were collected from active drinking water wells in the Greater Phoenix Area of Arizona. Sample 3 was collected from a discontinued drinking water well in Tucson, AZ, located approximately 120 m downgradient from a military base.^29^ Samples 4 and 5 were collected from extraction wells for pilot pump and treat systems at two different areas of the Naval Air Station Joint Reserve Base, Willow Grove, PA.^30^ Samples were collected using the clean protocol in EPA Method 533, to avoid contamination, and stored in 5 gallon, high-density polyethylene (HDPE) containers (Hedpak, EBK Containers, Lake in The Hills, IL) in the dark at 4 °C until analysis.

### 2.2 Chemicals and Reagents

PFAS standards (Table S1) were purchased from Wellington Laboratories (Guelph, ON, Canada). LC–MS grade ammonium acetate (99.0%) was obtained from Merck KGaA (Darmstadt, Germany). LC–MS-grade methanol was purchased from Millipore-Sigma (Burlington, MA). Ultrapure laboratory water (prepared using a Millipore IQ-7000 Milli-Q filtration system including 0.22 μm Q-POD, IPAK Meta, IPAK Quanta, and a LC-PAK filter for ultrapure water suitable for LC–MS, Bedford, MA) was used for chromatographic analysis in the mobile phase.

### 2.3 Sample Preparation

Groundwater samples were filtered through 0.22 μm polypropylene filters and transferred to Wheaton polypropylene HPLC vials (Millville, NJ) with polypropylene caps from Agilent Technologies (Santa Clara, CA) before injection into the HPLC-HRMS/MS system. Samples were analyzed using the direct injection method.

### 2.4 Liquid Chromatography Tandem Mass Spectrometry

Sample analysis was performed using an HPLC-HRMS/MS system, which consisted of an UltiMate 3000 Rapid Separation LC (RSLC) high-pressure liquid chromatography instrument from Dionex (Sunnyvale, CA) coupled to a Q-Exactive Focus Hybrid quadrupole-Orbitrap mass spectrometer from Thermo Fisher Scientific (San Jose, CA). Analyte chromatographic separation was achieved on a Gemini C18 analytical column (100 mm × 3.0 mm, 3 μm particle size) from Phenomenex (Torrance, CA) with an injection volume of 20 μL and a column oven temperature maintained at 40 °C. Samples were chromatographically separated with a 0.5 mL min^–1^ gradient of aqueous 20 mM ammonium acetate (mobile phase A) and methanol (mobile phase B) as follows: 0.0 min: 90% A, 1.0 min: 35% A, 18.0 min: 20% A, 18.1 min: 1% A, 22.0 min: 1% A, 22.3 min: 90% A, 25.3 min: 90% A.

Initial HRMS/MS parameters were based on Koronaiou et al.^31^ These parameters were then optimized using PFAS standards to improve the method sensitivity for the current study. The heated electrospray ionization source (HESI-II) was operated in negative ionization mode (ESI−) with a capillary potential of 3.0 kV, a capillary temperature of 300 °C, and a vaporization temperature of 500 °C. A DDA MS^2^ experiment in Discovery Mode (ddMS^2^) was utilized for suspect screening. High-purity nitrogen was used for the nebulizer and collision gases. Full scan mass spectra MS^1^ (precursor ion) (MS^1^, 70,000 mass resolution) and fragmentation spectra (MS^2^, 17,500 mass resolution) were recorded using DDA. A mass range of 70–1040 *m*/*z* (mass-to-charge ratio) was used for MS^1^. MS^2^ data were recorded with stepped collision energies of 15, 30, and 50 eV. Both MS^1^ and MS^2^ spectra were recorded as centroids. Additional HRMS parameters are listed in Table 1. DDA, which requires a tandem mass analyzer, employs a full scan (MS^1^) event to dynamically select the most abundant ions (precursors) for collisional-induced fragmentation, generating product ion spectra.^17^ Because this approach does not require a predefined target mass list, it enables the detection of unexpected precursor ions (PFAS) encountered in the MS^1^ chromatogram.

**Table 1:** Instrumental Parameters in Orbitrap ddMS^2^ Acquisition

HESI-II	
sheath gas flow rate	50
Aux gas flow rate	15
sweep gas flow rate	0
S-lens RF level	55
capillary temperature (°C)	300
vaporizer temperature (°C)	500
spray voltage (|kV|)	3.0

full MS	
polarity	negative
resolution	70,000
scan range	70–1040 *m*/*z*
AGC target	1e6
maximum IT	auto
microscan	1
spectrum data type	Centroid

ddMS^2^: discovery	
resolution	17,500
isolation window	1 *m*/*z*
NCE	15, 30, 50
AGC Target	2e5
maximum IT	auto
loop count	2
minimum AGC target	8e3
intensity threshold	auto
Apex trigger	2–5s
dynamic exclusion	auto
exclude isotopes	On
spectrum data type	Centroid

After the HRMS/MS parameters were optimized and the HPLC method was established, PFAS standards were used to evaluate the detection limit of each compound in the present method.

### 2.5 Quality Control

Although quantitative analysis and reaching the lowest possible limits of detection were not primary objectives of this investigation, measures of quality control similar to those used in targeted analyses were implemented (Table S2). For example, to eliminate all artifacts and false positives, masses detected in ultrapure laboratory water blank samples were manually eliminated from the *m*/*z* features detected in the groundwater samples during data processing. Polypropylene autosampler vials and caps were used to avoid sample loss on the glass surfaces. A mixture of 25 PFAS standards was used to ensure that the DDA suspect screening method was operating correctly, and instrument blank samples were created from mobile phase solvents and ultrapure water run alongside environmental samples to monitor potential carryover. After analysis of standards, three blank sample injections were made before the groundwater samples sequence. Each sample was injected in duplicate. The systematic changes in retention time (*t*_R_) among homologous series identified were also used to exclude false positive assignments.

### 2.6 Data Processing and Feature Identification

Two software programs, TraceFinder (TF) and FreeStyle (FS) (both from Thermo Fisher Scientific), were used to establish a suspect screening workflow. Data were first processed by TF using the target identification module incorporating a modified version of the NIST Suspect List of Possible PFAS (adapted for compatibility with TF and limited to compounds up to 1040 Da). This target identification step produced a list of MS^1^ features (from full scan data) with a mass error threshold of ≤ 5 ppm, a commonly accepted value, and sufficiently stringent criterion in Orbitrap HRMS-based screening workflows; ≤5 ppm threshold reflects typical mass accuracy achievable in this Orbitrap instrument and strikes a balance between minimizing false positives and maintaining adequate sensitivity for suspect identification.^25^

Additionally, in the TF method, feature selection required a minimum peak area of 100,000 and a TF default signal-to-noise ratio (S/N) threshold of ≥ 50 was used. Finally, if an MS^1^ feature matched one of the 25 PFAS reference standards used, its *t*_R_ was confirmed to be within 0.1 min of the standard. From this list of *m*/*z* ions identified as PFAS, features with MS^2^ spectra were selected for further processing. To confirm the presence of PFAS, MS^2^ information was manually assessed; each *m*/*z* ion first identified as PFAS in the TF was examined in the FS to inspect the fragmentation spectra. To confirm a structure, MS^2^ fragmentation patterns were compared against reference spectra from mzCloud, PubChem, and previously published literature. When reference spectra were not available, fragmentation was further interpreted based on deduced structures produced in ChemDraw using theoretical molecular formulas, theoretical *m*/*z* fragment ions, and homology series information from Kendrick Mass Defect (KMD) calculations. All steps involved in the data processing workflow are summarized in Figure 1.

**Figure 1:**
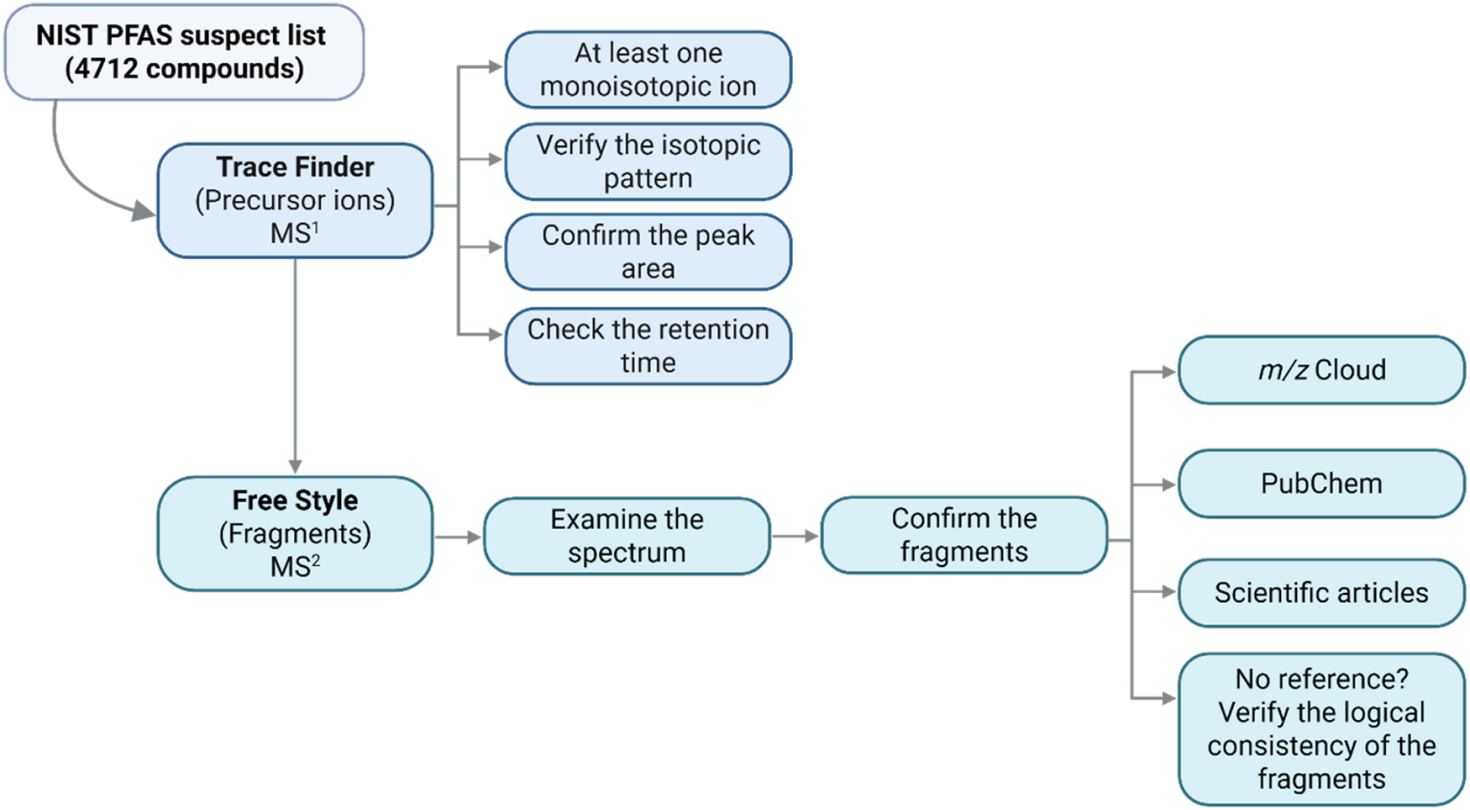
Suspect screening workflow.

Confidence levels (CL) were assigned according to a recently established PFAS identification scale, which ranges from Level 1 to Level 5. In summary, Level 1 indicates identification confirmed by analytical standard, Level 2 represents probable structures supported by library spectra matching or diagnostic fragments evidence, Level 3 corresponds to tentative candidates that have possible isomers, Level 4 can be assigned an unambiguous formula (but with multiple possible candidates), and Level 5 represents identification based solely on accurate mass without structural or formulaic information.^32^ Herein, only *m*/*z* features identified as PFAS that also exhibited MS^2^ fragmentation were considered, and all assigned confidence levels were attributed to either Level 1 or Level 2, because the observed fragmentation patterns were diagnostic and consistent with reported literature or reference standards, and the likelihood of coeluting structural isomers (Level 3) producing indistinguishable spectra under the applied chromatographic conditions was considered low.

To facilitate comparison among homologous compounds, the KMD was calculated for all detected features using CF_2_-normalized values, based on eqs 1 and 2.^33^ The resulting KMD plot, which displays the *m*/*z* versus the CF_2_-based mass defect, enables the visualization of homologous series, each exhibiting a characteristic and consistent mass defect. All confirmed compounds were evaluated using this method to support their classification within the PFAS series.

1 $$\;Kendrick\;mass\;\left(KM\right)=measured\;mass\times \frac{no\min al\;mass\;of\;{CF}_{2}}{exact\;mass\;of\;{CF}_{2}}$$

2 Kendrickmassdefect(KMD)=nominalmass(round)−Kendrickmass(KM)

## 3 Results and Discussion

First, after the HPLC-HRMS/MS method parameters were established, PFAS standards were analyzed to validate the suspect screening method. Detection limits obtained are presented in Table S2 and confirm the method’s ability to reliably detect PFAS. Then, the five groundwater samples were analyzed by the established suspect screening method.

After DDA MS^2^ in discovery mode, data analysis began by using TF software with a suspect screening list comprising 4712 per- and polyfluoroalkyl substances obtained from the NIST database.^34^ Initially, TF was employed for MS^1^ screening based on *m*/*z* values. To be considered a valid detection, at least one monoisotopic ion had to be present, with peak area integrated, detected in both replicates with S/N ≥ 50, and absent in the blank sample (Milli-Q water analyzed under identical conditions). Following this initial precursor ion screen, the reduced data set was evaluated in FS to examine product ions, enabling the assessment of fragmentation patterns and comparison with spectra from external libraries, including “m/z Cloud Advanced Mass Spectral Database”^35^ and PubChem.^36^

In total, 11 PFAS were identified with CL 1 or 2. Six compounds were detected in Sample 3, two compounds in Sample 4, and 11 PFAS in Sample 5. No PFAS were detected in samples 1 and 2. A summary of all suspect compounds detected in this study, including their acronyms, class, molecular formulas, *m*/*z*, *t*_R_, mass accuracy (Δ*m*/*z*), and assigned CL, is provided in Table 2. The corresponding chemical structures and CAS numbers are available in Table S3 in the Supporting Information.

**Table 2:** Identification of PFAS from Suspect Screening in Samples 3, 4, and 5, Including Compound Class, Molecular Formula, Theoretical *m*/*z*, *t*_R_, Mass Accuracy (Δ*m*/*z*), and CL for Each Compound

samples	compounds	class	molecular formula	theoretical [M – H]^−^	*t*_R_	delta *m*/*z* (ppm)	CL^a^
3	PFBS	PFSA	C_4_HF_9_O_3_S	298.943	3.8	0.8273	1
	PFPeS	PFSA	C_5_HF_11_O_3_S	348.9398	4.3	0.9737	1
	PFHxS	PFSA	C_6_HF_13_O_3_S	398.9366	4.8	1.007	1
	FBSA	FASA	C_4_H_2_F_9_NO_2_S	297.959	4.1	–0.139	2
	FHxSA	FASA	C_6_H_2_F_13_NO_2_S	397.9526	5.5	0.8843	2
	1*H*-perfluoro-pentane	perfluoroalkanes	C_5_HF_11_	268.983	4.3	0.9884	2
4	PFHxS	PFSA	C_6_HF_13_O_3_S	398.9366	4.8	0.7392	1
	1*H*-perfluoro-heptane	perfluoroalkanes	C_7_HF_15_	368.9766	5.6	0.6428	2
5	PFBS	PFSA	C_4_HF_9_O_3_S	298.943	3.8	–0.1936	1
	PFPeS	PFSA	C_5_HF_11_O_3_S	348.9398	4.3	0.3178	1
	PFHxS	PFSA	C_6_HF_13_O_3_S	398.9366	4.8	0.0125	1
	PFOS	PFSA	C_8_HF_17_O_3_S	498.9302	6.6	0.4925	1
	6:2 FTS	FTS	C_8_H_5_F_13_O_3_S	426.9679	5.5	0.2877	1
	FPrSA	FASA	C_3_H_2_F_7_NO_2_S	247.9622	3.6	–0.8476	2
	PFPrS	PFSA	C_3_HF_7_O_3_S	248.9462	3.4	–0.7054	2
	FBSA	FASA	C_4_H_2_F_9_NO_2_S	297.959	4.1	–0.139	2
	1*H*-perfluoro-heptane	perfluoroalkanes	C_7_HF_16_	368.9766	5.6	0.1879	2
	FHxSA	FASA	C_6_H_2_F_13_NO_2_S	397.9526	5.5	–0.036	2
	1*H*-perfluoro-pentane	perfluoroalkanes	C_5_HF_12_	268.983	4.3	0.1374	2

a CL, Confidence Level; CL 1 confirmed using analytical standard, CL 2 Discovery.

Figure 2 displays the CF_2_ KMD plot of all 11 ions identified by the suspect screening workflow. Homologous PFAS series sharing the same KMD align horizontally in the plot, confirming that the compounds belong to the same class. The *m*/*z* vs KMD (CF_2_) plot revealed three homologous series (with ≥2 members) of PFAS in the groundwater sample(s): perfluoroalkanesulfonic acids (PFSA), perfluoroalkanesulfonamides (FASA), and perfluoroalkanes. The fourth series, fluorotelomer sulfonic acids (FTS), is represented by only one identified compound.

**Figure 2:**
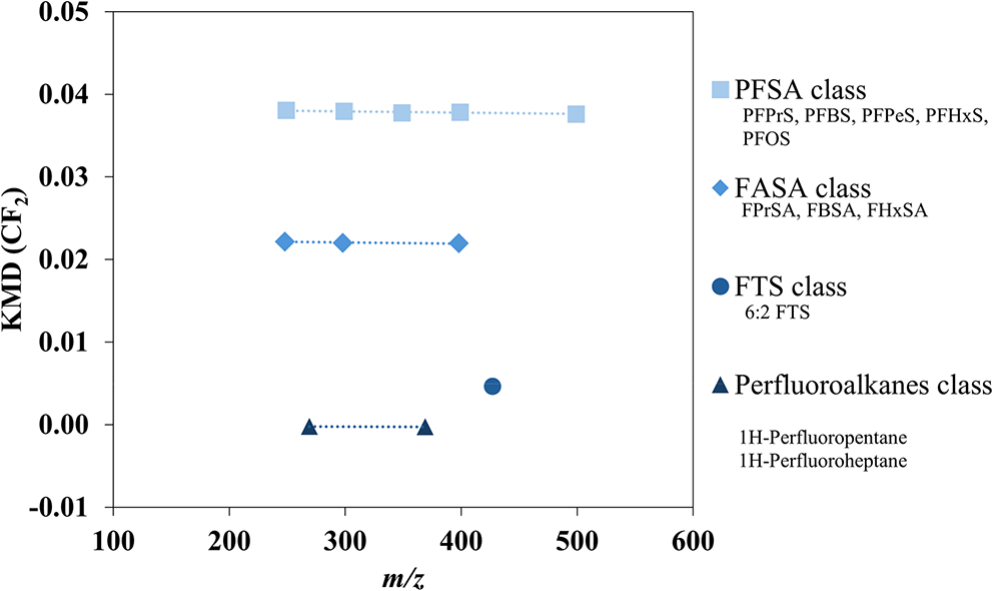
CF_2_ mass defect plot for four detected PFAS classes, including all identified PFAS. Solid symbols aligned horizontally indicate four PFAS classes: PFSA, FASA, FTS, and perfluoroalkanes. The dashed lines represent the homologous series (three in total).

PFHxS was detected in Sample 3 (Tucson) and in Willow Grove Samples 4 and 5. PFBS and perfluoropentanesulfonic acid (PFPeS) were detected in Samples 3 and 5, while 6:2 fluorotelomer sulfonic acid (6:2 FTS) and PFOS were exclusively identified in Sample 5. All were confirmed with CL 1, based on *t*_R_ and MS^2^ fragmentation matched with certified reference standards.

For PFBS, a characteristic PFSA fragmentation pattern containing two fragment ions (FO_3_S^–^, SO_3_^–^) was detected from the precursor ion *m*/*z* = 298.9430, matching the same pattern in the standard, and with a *t*_R_ difference of only 0.04 min (Figure S1). PFPeS exhibited the same PFSA fragmentation pattern, with three fragment ions (C_2_F_5_^–^, FO_3_S^–^, and SO_3_^–^) from precursor ion *m*/*z* = 348.9398, consistent with the standard and a Δ*t*_R_ of 0.03 min (Figure S2). PFHxS, present in samples 3, 4, and 5, also showed the same PFSA fragmentation pattern, with three fragment ions (C_3_F_7_^–^, FO_3_S^–^, and SO_3_^–^) arising from precursor ion *m*/*z* = 398.9366, with Δ*t*_R_ = 0.04 min (Figure S3). And finally, PFOS was confirmed with the same PFSA fragmentation pattern, with three fragments (C_8_F_17_^–^, FO_3_S^–^, SO_3_^–^) from precursor ion *m*/*z* = 498.9302 with acceptable mass defect (<5 ppm) and Δ*t*_R_ = 0.08 min (Figure S4). The 6:2 FTS fragmentation spectrum revealed multiple characteristic fragments (C_8_H_3_F_12_O_3_S^–^, C_8_H_2_F_11_O_3_S^–^, C_7_F_11_^–^, C_4_H_4_O_2_F_3_^–^, HO_3_S^–^, and SO_3_^–^), all consistent with the standard and Δ*t*_R_ = 0.07 min (Figure S5).

All five of these compounds were previously reported from the same groundwater grab samples by Menezes et al., who applied an LC–MS/MS MRM method targeting 25 PFAS, as well as the TOP assay and TOF quantification via CIC. In their study, the sum of the targeted PFAS quantified (∑PFAS(25)) accounted for 41.7%, 92.8%, and 67.8% of the TOF in Samples 3, 4, and 5, respectively, highlighting the fraction of organofluorine not explained by the targeted analysis.^10^ Our identification of five PFAS (PFBS, PFPeS, PFHxS, PFOS, and 6:2 FTS) in the same samples, compounds also detected by Menezes et al. using targeted LC–MS/MS, primarily serves to validate the operability of our suspect screening workflow.

More importantly, the greater value of this approach lies in its ability to identify previously unrecognized PFAS that are not captured by targeted methods. Based on a comparison of TOF with the PFAS quantified by Menezes et al., newly identified, untargeted PFAS detected here by suspect screening could account for up to 58.3%, 7.2%, and 32.2% of TOF in Samples 3, 4, and 5, respectively.^10^ Because the suspect screening method used in this study is qualitative, quantitative estimates cannot be derived directly. Nevertheless, when combined with the data reported by Menezes et al., our results demonstrate that at least a portion of the remaining organofluorine corresponds to PFAS outside the scope of the targeted methods. Six such PFAS were identified in this work: perfluoropropanesulfonamide (FPrSA), perfluoropropanesulfonic acid (PFPrS), perfluorobutylsulfonamide (FBSA), 1*H*-perfluoroheptane, perfluorohexanesulfonamide (FHxSA), and 1*H*-perfluoropentane. Thus, the combined application of complementary PFAS measurement strategies, suspect screening, targeted LC–MS/MS, and TOF by CIC begins to enumerate additional PFAS overlooked by targeted analyses. Using the DDA-based screening method, six previously unreported and nontarget PFAS were identified in the same groundwater samples.

FPrSA was identified with CL 2 in Sample 5. As shown in Figure 3, the fragmentation spectrum displayed three characteristic FASA fragment ions: C_3_F_7_^–^ (*m*/*z* = 168.9885), FO_2_S^–^ (*m*/*z* = 82.9595), and NO_2_S^–^ (*m*/*z* = 77.9641). Two of these fragments supported the proposed structure, consistent with annotations by Dewapriya et al. in their nontarget and suspect screening work on blood and serum samples from cattle exposed to AFFF-contaminated groundwater.^37^

**Figure 3:**
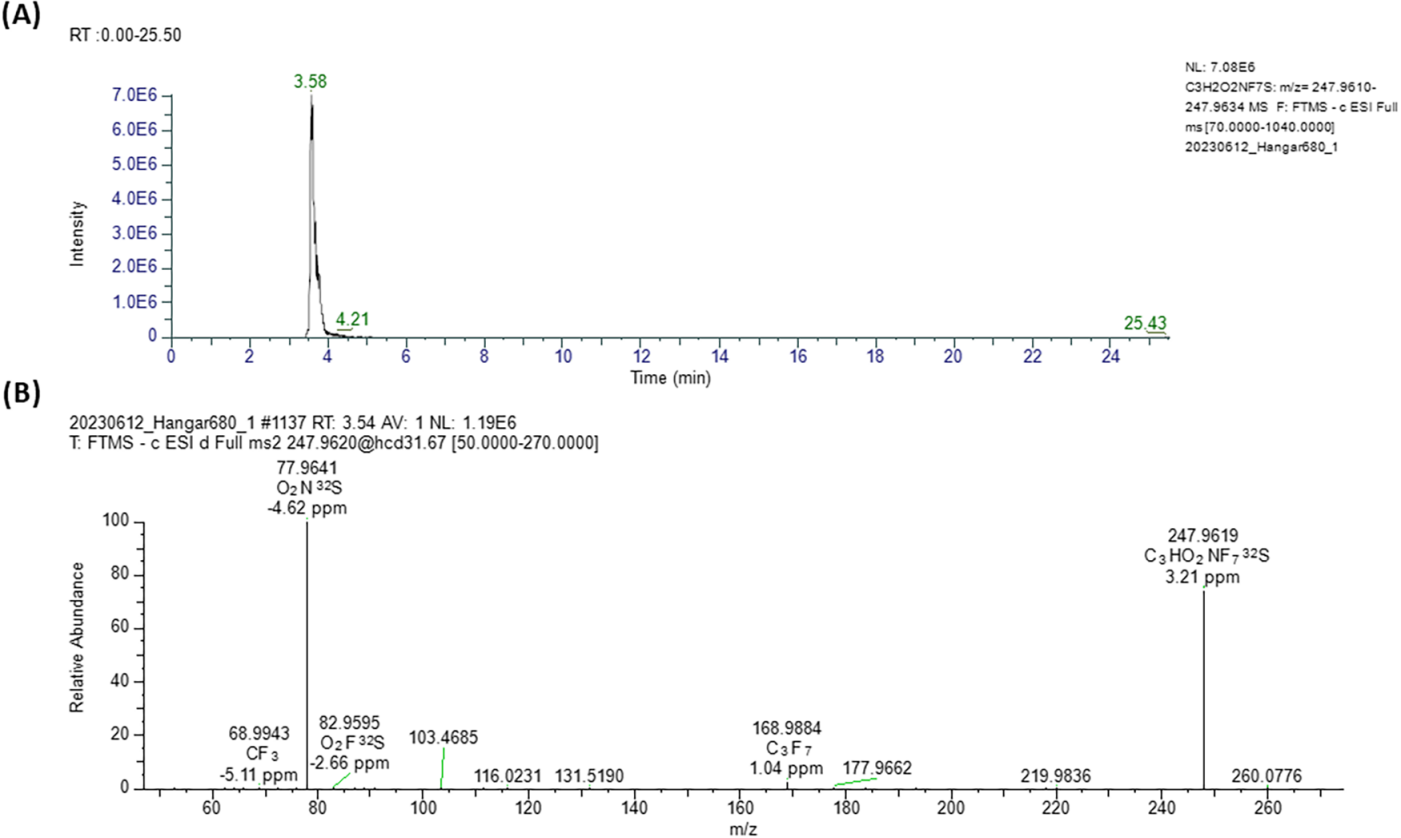
Chromatographic peak in extracted ion chromatogram (A) and the corresponding fragmentation (MS^2^) spectrum for FPrSA at *t*_R_ 3.54 min (B) in Sample 5. The MS^2^ spectrum displays three typical FASA fragment ions: C_3_F_7_^–^ (*m*/*z* = 168.9884), FO_2_S^–^ (*m*/*z* = 82.9595), and NO_2_S^–^ (*m*/*z* = 77.9641).

As shown in Figure 3 (and the other spectra in Supporting Information), all MS^1^ data were obtained using TF, while MS^2^ data were processed and verified in FS. Therefore, minor discrepancies may exist between exact mass values reported by each software due to differences in the number of decimal places considered. However, all values remained within the accepted mass error tolerance of ± 5 ppm.

Another compound identified with CL 2 was PFPrS detected in Sample 5 (Figure S6), eluting at 3.39 min. The MS^2^ spectrum showed three characteristic FASA fragments: C_2_F_5_^–^ (*m*/*z* = 118.9911), FO_3_S^–^ (*m*/*z* = 98.9544), and O_3_S^–^ (*m*/*z* = 79.9559). These ions are consistent with the proposed structure and with fragmentation patterns previously reported in the literature.^38^ Due to its high water solubility, short-chain PFPrS is frequently detected in aqueous matrices.^39^ For instance, Mak et al. reported PFPrS using a targeted method in Japanese tap water samples collected between 2006 and 2008.^40^ Barzen-Hanson and Field also found it in all 11 groundwater samples tested from US military bases using a nontarget method with Data-Independent Acquisition (DIA).^19^ Wu et al. also confirmed its presence in multiple AFFF formulations via suspect screening.^38^

Using additional resources such as the mzCloud^35^ spectral library, FBSA was identified in Samples 3 and 5 with CL 2 (Figure S7). The MS^2^ spectrum showed multiple fragments supporting the proposed structure, including C_4_F_9_^–^ (*m*/*z* = 218.9857), C_2_F_5_^–^ (*m*/*z* = 118.9915), and NO_2_S^–^ (*m*/*z* = 77.9641). FBSA has been reported in various matrices: it has been quantified in biota,^41^ detected in blood and serum of cattle exposed to AFFF-contaminated groundwater via suspect screening,^37^ and identified in effluents from semiconductor manufacturing using a nontarget method.^42^

1*H*-Perfluoroheptane was detected in Samples 4 and 5 as a Level 2 compound, eluting at 5.6 min (Figure S8). The MS^2^ spectrum revealed fragments typical of PFAS: C_4_F_9_^–^ (*m*/*z* = 218.9857), C_3_F_7_^–^ (*m*/*z* = 168.9884), and C_2_F_5_^–^ (*m*/*z* = 118.9912). These fragments support its identification, which is based on known logical fragmentation pathways for perfluoroalkyl compounds.

FHxSA was also identified in Samples 3 and 5 with CL 2, supported by comparison with fragmentation data from Dewapriya et al.^37^ The MS^2^ spectrum revealed at least three characteristic FASA fragments: C_6_F_13_^–^ (*m*/*z* = 318.9791), C_4_F_9_^–^ (*m*/*z* = 218.9848), and NO_2_S^–^ (*m*/*z* = 77.9641) (Figure S9). FHxSA has been detected in AFFF-impacted groundwater using a targeted method^43^ and in cattle serum exposed to contaminated environments via suspect screening.^37^

1*H*-Perfluoropentane was detected in Samples 3 and 5 and with CL 2 (Figure S10). The MS^2^ spectrum exhibited two major fragments: C_2_F_5_^–^ (*m*/*z* = 118.9912) and CF_3_^–^ (*m*/*z* = 68.9943), consistent with the expected fragmentation of this structure. As with 1*H*-Perfluoroheptane, these ions support structural assignment based on logical fragmentation. Previous study quantified 1*H*-Perfluoroheptane and 1*H*-Perfluoropentane in the emissions from AFFF incineration.^44^

A summary of all fragment ions reported for each identified compound, including their accurate masses, mass errors, and supporting identification references, is compiled in Table S4.

Nontarget analysis has expanded rapidly due to the increasing availability of HRMS/MS and advanced data processing tools. Publications over the past two decades reflect this technology growth.^45^ Today, advanced software is designed to maximize annotation confidence for unknown PFAS, although complex and computationally intensive data processing remains common. Recent studies have reported several hundred PFAS, spanning more than 40 structural classes, from extracts of AFFF-impacted groundwater analyzed by LC coupled to quadrupole time-of-flight (QToF) mass spectrometry, using both vendor-provided and third-party software tools.^22,27,28^ When samples were extracted using liquid or passive-sampler techniques, the number of PFAS identified was eight to 20 times greater than in our results. The larger number of PFAS reported in these studies (25–240 compounds) is also attributable to the data-acquisition strategies employed. In two of these works, both positive- and negative-mode ESI were used, enabling the detection of PFAS not detectable by targeted negative-mode methods. Both suspect screening and nontarget analysis workflows were used in combination in the studies reporting the highest number of PFAS identifications. In one case, each sample was analyzed twice using complementary acquisition strategies, with data-independent acquisition (DIA) used to prioritize features, followed by DDA acquisition for compound identifications. In addition, the greater PFAS coverage achieved by suspect screening in two of these studies was supported by in-house, curated spectral libraries built from the literature and measurements of neat AFFF material, as well as, in one recent effort, a custom Python-based feature-prioritization tool. The studies reporting the highest number of PFAS also analyzed more groundwater samples (10 and 13 samples), which likely contributed to their higher PFAS counts. Overall, workflows with greater analytical complexity, including advanced software, curated spectral libraries, multiple acquisition modes, and extensive sample preparation, tend to generate more features and, consequently, more identified PFAS across diverse structural classes. In contrast, direct injection combined with a simplified suspect screening approach, such as the TF and FS workflow applied here, inherently yields fewer PFAS identifications due to reduced sensitivity and lower analytical coverage. Sample preconcentration by extraction considerably increases the likelihood of detecting PFAS that may be missed under direct-injection conditions.

Although the total number of PFAS identified in the present study is smaller, the proposed workflow is more accessible to a broader range of laboratories and therefore represents a valuable and practical tool. Importantly, this approach enabled the identification of short-chain (<C6) and ultrashort-chain (<C3) PFAS, which is particularly relevant given the ongoing industrial transition toward these compounds as alternatives to legacy PFAS and the limited number of environmental studies addressing their occurrence and behavior. Short-chain PFAS are highly water-soluble, exhibit low to moderate sorption to soils and sediments, and are resistant to biological and chemical degradation, factors that contribute to their widespread occurrence in aquatic environments.^46^ Moreover, remediation of these compounds remains challenging, as conventional ex situ adsorption technologies (e.g., granular activated carbon) often show poor performance due to early breakthrough of these highly soluble species.^19^

## 4 Conclusions

This study successfully employed a nontarget analytical workflow combining HRMS/MS and suspect screening tools to characterize PFAS in groundwater samples contaminated by AFFF. Identification of six nontarget PFAS (1*H*-perfluoropentane, 1*H*-perfluoroheptane, FBSA, FHxSA, FPrSA, and PFPrS) revealed the presence of more complex PFAS mixtures that classical targeted analyses missed in the same groundwater samples. These results highlight the necessity of additional analytical tools and strategies for a more comprehensive understanding of PFAS contamination. We showed that a modest suspect screening workflow, without the need for advanced software, is sufficient to identify nontarget PFAS and effectively lower the nontarget PFAS discovery barrier for more laboratories.

The openly available NIST Suspect List of Possible PFAS, comprising approximately 4700 PFAS, was employed with TraceFinder software for rapid screening based on exact mass matching. This allowed for the detection of additional PFAS not captured by targeted approaches. Characteristic fragmentation patterns for PFSA, including FO_3_S^–^ and SO_3_^–^ ions, were repeatedly observed in the FreeStyle workflow step, pointing toward several PFSA identifications. The use of FreeStyle software (available at no cost to Orbitrap instrument users) to manage measured product ion spectra, and comparative spectra published in collections like mzCloud and PubChem, opens up opportunities for more laboratories to undertake PFAS suspect screening investigations. The accessible workflow developed in this study proved effective for the structural characterization of PFAS without incurring the cost of more powerful software, and without requiring authentic standards for an impossibly long list of known PFAS analytes. However, since direct injection was used in the liquid chromatography step, method sensitivity limitations typically observed when concentration and extraction are not used, likely prevented the detection of PFAS precursor ions (and accordingly product ions) present at lower concentrations. To address this limitation, a preconcentration step was later integrated into the experimental method to improve detection limits and enable even more analytical coverage of the same groundwater samples. These data are currently under analysis.

Our results emphasize that AFFF-impacted groundwater contains a variety of legacy and emerging PFAS, many of which may persist in the environment and potentially pose risks to aquatic ecosystems and human health through bioaccumulation and drinking water exposure. Therefore, the combination of targeted and nontarget HRMS/MS-based screening approaches is critical for comprehensive PFAS monitoring, environmental risk assessment, and the development of more informed regulatory and remediation strategies. The accessible workflow presented here can encourage further investigations by more users to assess the different aspects of PFAS contamination, e.g., mobility, fate, and toxicity of lesser-known (nontarget) PFAS.

## Supplementary Material



## Notes

Information about the PFAS standards used in the study (Table S1); the corresponding instrument LODs of each PFAS standard in the established LC-HRMS/MS method (Table S2); the corresponding chemical structures and CAS numbers of all PFAS identified in the study (Table S3); a summary of observed fragment ions for the identified PFAS compounds (Table S4); all chromatograms and spectra for each identified compound (Figures S1–S10) (PDF)

## Abbreviations

AFFF: aqueous film-forming foam
CIC: combustion ion chromatography
CL: confidence level
DDA: data-dependent acquisitions
EPA: Environmental Protection Agency
FBSA: perfluorobutylsulfonamide
FHxSA: perfluorohexanesulfonamide
FS: FreeStyle
FPrSA: perfluoropropanesulfonamide
HPLC-HRMS/MS: liquid chromatography coupled with Orbitrap high resolution tandem mass spectrometry
LC–MS: liquid chromatography–mass spectrometry
MS^1^: precursor ion
MS^2^: product ions
PFOA: perfluorooctanoic acid
PFBS: perfluorobutanesulfonic acid
PFHxS: perfluorohexanesulfonic acid
PFNA: perfluorononanoic acid
PFOS: perfluorooctanesulfonic acid
PFPeS: perfluoropentanesulfonic acid
PFPrS: perfluoropropanesulfonic acid
PFAS: Per- and polyfluoroalkyl substances
TF: TraceFinder (TF)
TOF: total organic fluorine
TOP: total oxidizable precursor
*t*_R_: retention time
6:2 FTS: 6:2 fluorotelomer sulfonic acid
