# Supplementary material for: Suspect Screening for PFAS in Groundwater with an Accessible LC–MS Workflow
Source: ACS Omega. 2026 Mar 20;11(13):20145–54. doi: 10.1021/acsomega.5c08713 (PMC13063162; doi:10.1021/acsomega.5c08713)
Supplement: Supplementary file 1 [file ao5c08713_si_001.pdf]

Supporting Information

Suspect screening for PFAS in groundwater with an accessible LC-MS workflow

Bianca F. da Silva[1]; Kya N. Bruckner[2], Sonia C. N. Queiroz[3]; Carla B. G. Bottoli1[1]; Jon Chorover[2]; Leif Abrell[2]*

[1] Institute of Chemistry, National Institute of Science and Technology of Bioanalytics Lauro Kubota, Universidade Estadual de Campinas, Cidade Universitária Zeferino Vaz, 13083-970 Campinas, SP, Brazil.

[2] Department of Environmental Science, The University of Arizona, 85721, Tucson, AZ, USA

[3] Embrapa Meio Ambiente, Laboratório de Resíduos e Contaminantes, Rodovia SP 340, km 127.5, 13918-110, Jaguariúna, SP, Brazil

Table S1. PFAS analytes (n=25) used for identification, along with their acronyms, molecular formula, and CAS registry numbers. All standards used in this study were procured from *Wellington Laboratories*.

PFAS	Acronym	CAS ID	Molecular Formula
**Perfluoroalkyl Carboxylates**			
Perfluorobutanoic acid	PFBA	375-22-4	$C_4HF_7O_2$
Perfluoropentanoic acid	PFPeA	2706-90-3	$C_5HF_9O_2$
Perfluorohexanoic acid	PFHxA	307-24-4	$C_6HF_{11}O_2$
Perfluoroheptanoic acid	PFHpA	375-85-9	$C_7HF_{13}O_2$
Perfluorooctanoic acid	PFOA	335-67-1	$C_8HF_{15}O_2$
Perfluorononanoic acid	PFNA	375-95-1	$C_9HF_{17}O_2$
Perfluorodecanoic acid	PFDA	335-76-2	$C_{10}HF_{19}O_2$
Perfluoroundecanoic acid	PFUnA	2058-94-8	$C_{11}HF_{21}O_2$
Perfluorododecanoic acid	PFDoA	307-55-1	$C_{12}HF_{23}O_2$
Perfluorotridecanoic acid	PFTriDA	72629-94-8	$C_{13}HF_{25}O_2$
Perfluorotetradecanoic acid	PFTreA	376-06-7	$C_{14}HF_{27}O_2$
**Perfluoroalkyl sulfonates**			
Perfluorobutanesulfonic acid	PFBS	375-73-5	$C_4HF_9O_3S$
Perfluoropentanesulfonic acid	PFPeS	2706-91-4	$C_5HF_{11}O_3S$
Perfluorohexanesulfonic acid	PFHxS	355-46-4	$C_6HF_{13}O_3S$
Perfluoroheptanesulfonic acid	PFHpS	375-92-8	$C_7HF_{15}O_3S$
Perfluorooctanesulfonic acid	PFOS	1763-23-1	$C_8HF_{17}O_3S$
Perfluorononanesulfonic acid	PFNS	68259-12-1	$C_9HF_{19}O_3S$
Perfluorodecanesulfonic acid	PFDS	335-77-3	$C_{10}HF_{21}O_3S$
**Perfluoroalkyl sulfonamides**			
Perfluorooctanesulfonamide	FOSA	754-91-6	$C_8H_2F_{17}NO_2S$
N-methylperfluorooctanesulfonamide	N-MeFOSA	31506-32-8	$C_9H_4F_{17}NO_2S$
2-(N-Methylperfluorooctanesulfonamido) acetic acid	NMeFOSAA	2355-31-9	$C_{11}H_6F_{17}NO_4S$
2-(N-Ethylperfluorooctanesulfonamido) acetic acid	NEtFOSAA	2991-50-6	$C_{12}H_8F_{17}NO_4S$
**Fluorotelomer sulfonates**			
Fluorotelomer sulphonic acid 4:2	4:2 FTS	757124-72-4	$C_6H_5F_9O_3S$
Fluorotelomer sulphonic acid 6:2	6:2 FTS	27619-97-2	$C_8H_5F_{13}O_3S$
Fluorotelomer sulphonic acid 8:2	8:2 FTS	39108-34-4	$C_{10}H_5F_{17}O_3S$

Table S2. Evaluation of the detection limits of each PFAS standard in the established LC-HRMS/MS method.

PFAS	LOD (μg L^{-1})
**Perfluoroalkyl Carboxylates**	
PFBA	1.0
PFPeA	0.01
PFHxA	0.1
PFHpA	0.1
PFOA	10.0
PFNA	0.01
PFDA	0.1
PFUnA	1.0
PFDoA	1.0
PFTriDA	1.0
PFTreA	1.0
**Perfluoroalkyl sulfonates**	
PFBS	0.01
PFPeS	0.01
PFHxS	0.01
PFHpS	0.01
PFOS	0.1
PFNS	0.1
PFDS	0.1
**Perfluoroalkyl sulfonamides**	
FOSA	0.1
N-MeFOSA	0.1
NMeFOSAA	1.0
NEtFOSAA	1.0
**Fluorotelomer sulfonates**	
4:2 FTS	0.1
6:2 FTS	0.1
8:2 FTS	0.1

Table S3. Identification of PFAS compounds from suspect screening including compound acronym, class, and their chemical structure. Assignments are categorized as Confirmed using analytical standards (CL 1) and Discovery (CL 2).

	Compounds	CAS number	Class	Structure
Confirmed using analytical standards	PFBS	375-73-5	PFSA	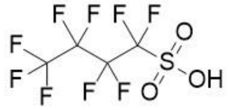
	PFPeS	2706-91-4	PFSA	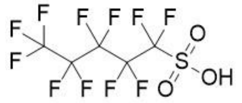
	PFHxS	355-46-4	PFSA	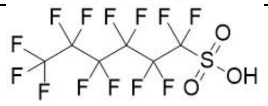
	PFOS	1763-23-1	PFSA	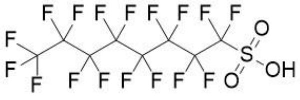
	6:2 FTS	27619-97-2	n:2 FTSA	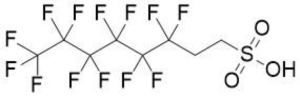
Discovery	FPrSA	152894-03-6	FASA	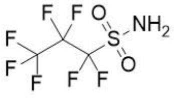
	PFPrS	423-41-6	PFSA	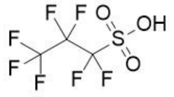
	FBSA	30334-69-1	FASA	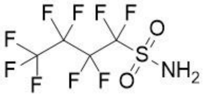
	1*H*-Perfluoroheptane	375-83-7	Perfluoroalkanes	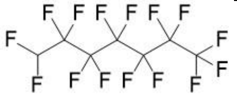
	FHxSA	41997-13-1	FASA	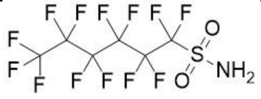
	1*H*-Perfluoropentane	375-61-1	Perfluoroalkanes	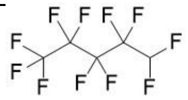

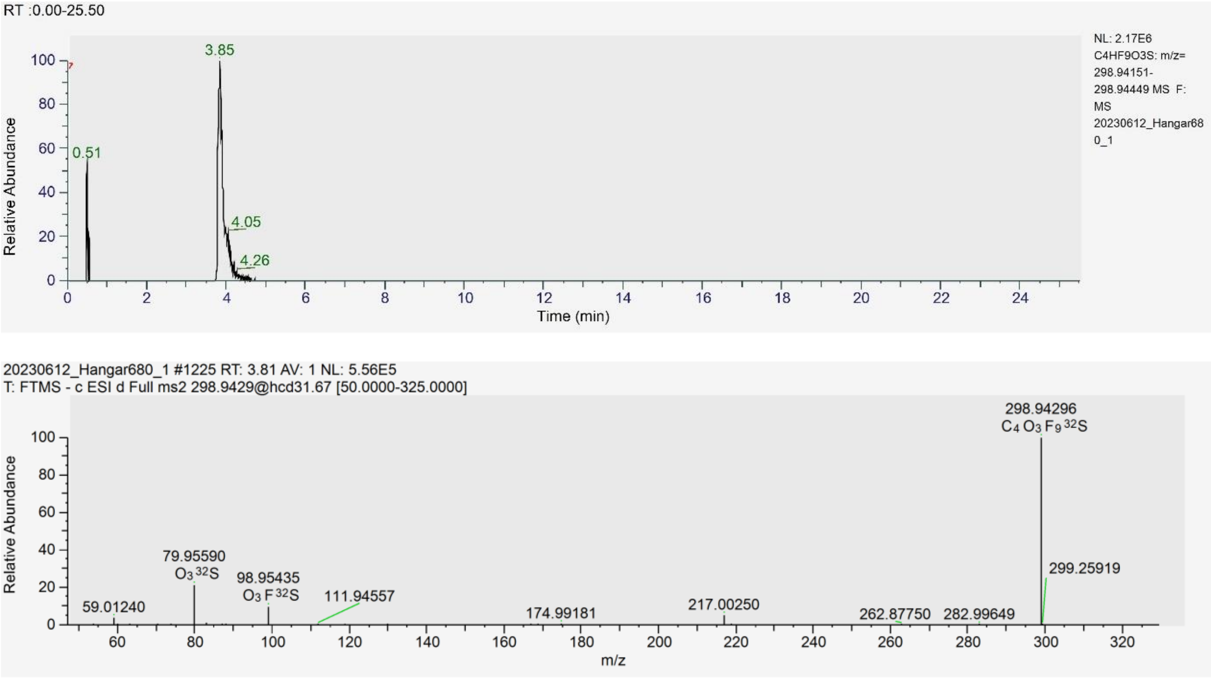

Standard

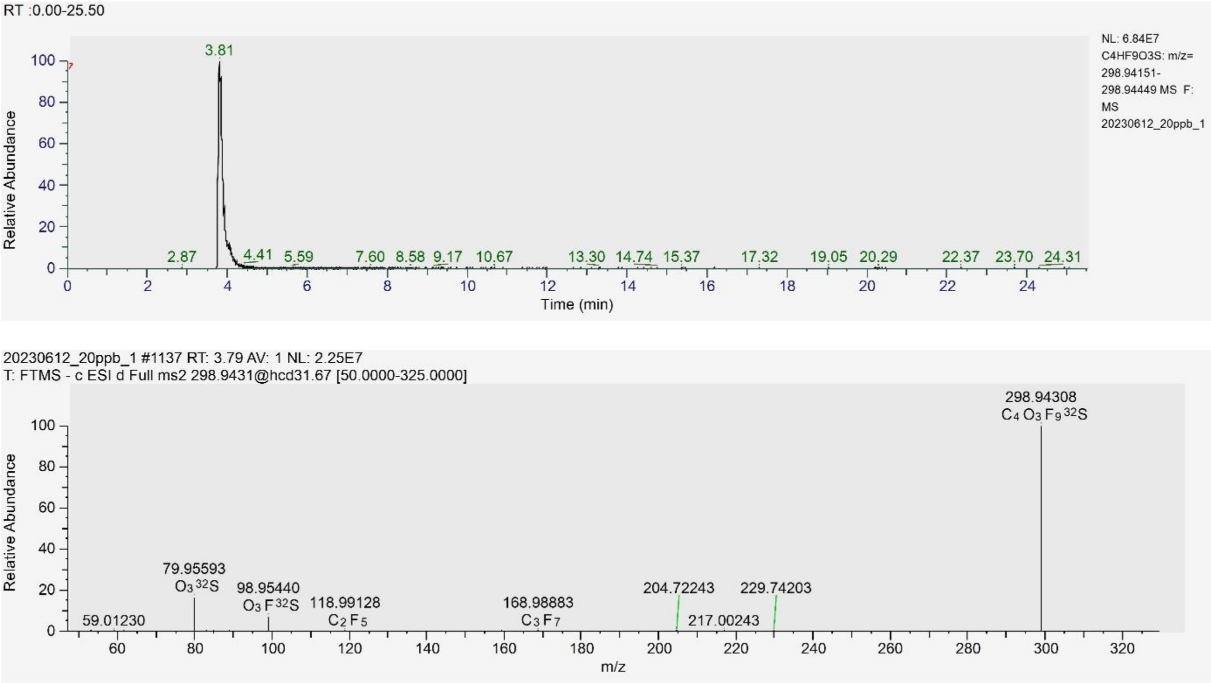

Figure S1. Confirmation of **PFBS** identification in Sample 5 (top) by comparison with reference standard (bottom). Retention times match (0.04 min) as well as the dominant fragments in the spectrum.

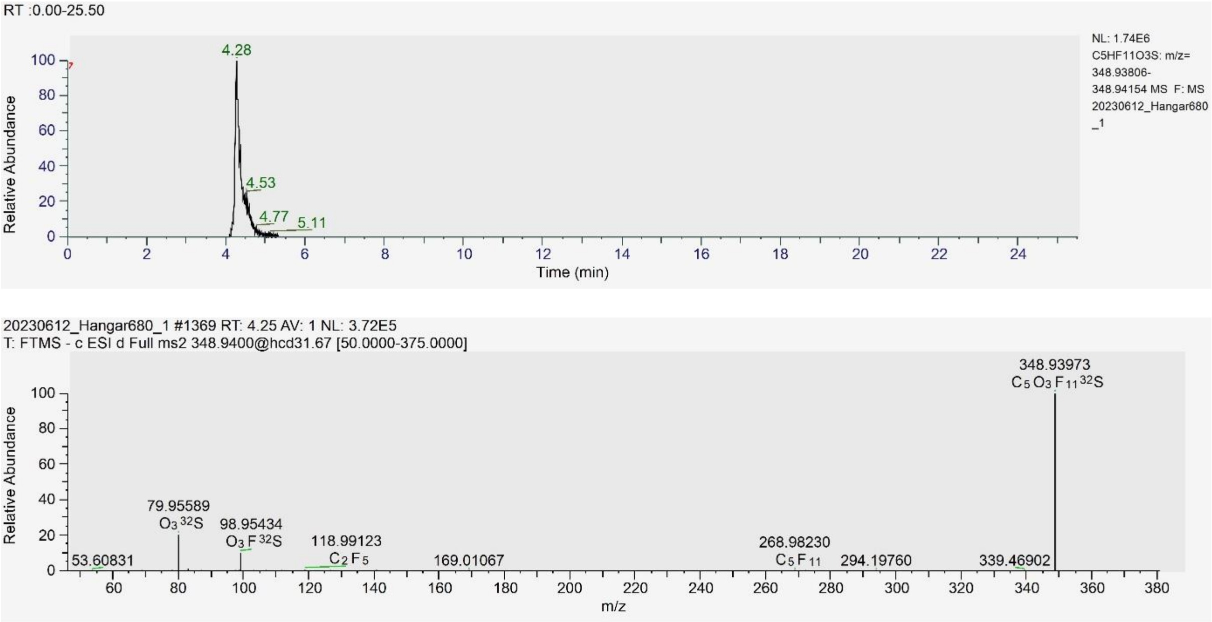

Standard

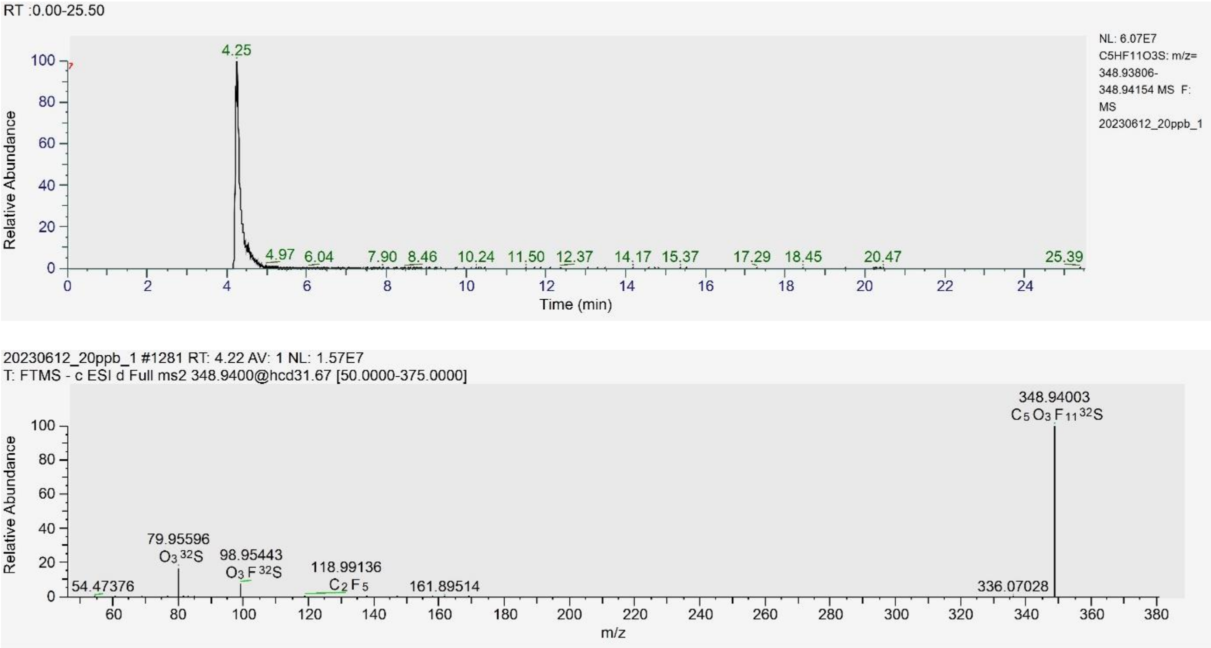

Figure S2. Confirmation of **PFPeS** identification in Sample 5 (top) by comparison with reference standard (bottom). Retention times match (0.03 min) as well as the dominant fragments in the spectrum.

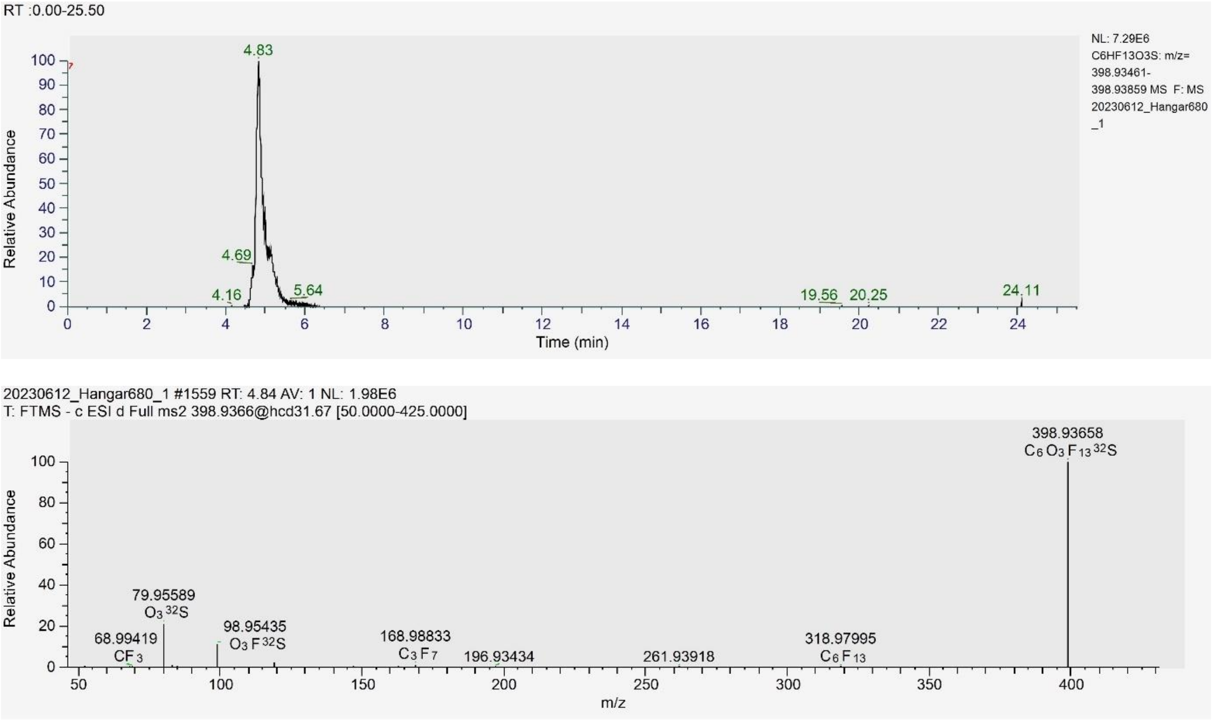

Standard

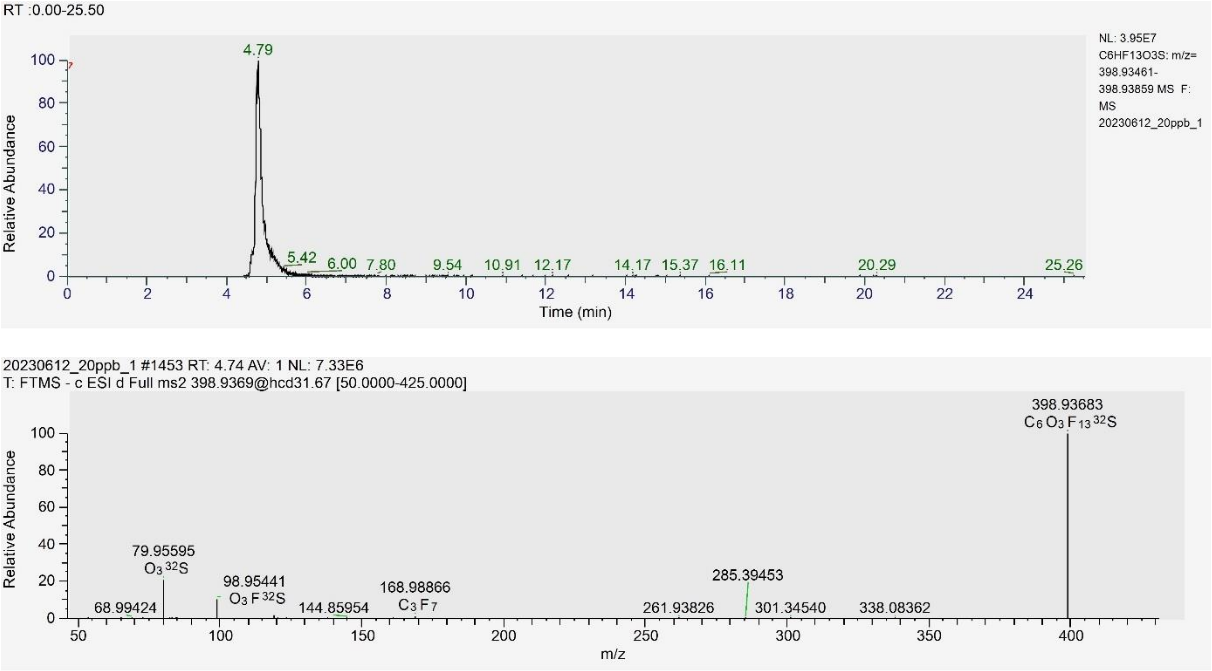

Figure S3. Confirmation of **PFHxS** identification in Sample 5 (top) by comparison with reference standard (bottom). Retention times match (0.04 min) as well as the dominant fragments in the spectrum.

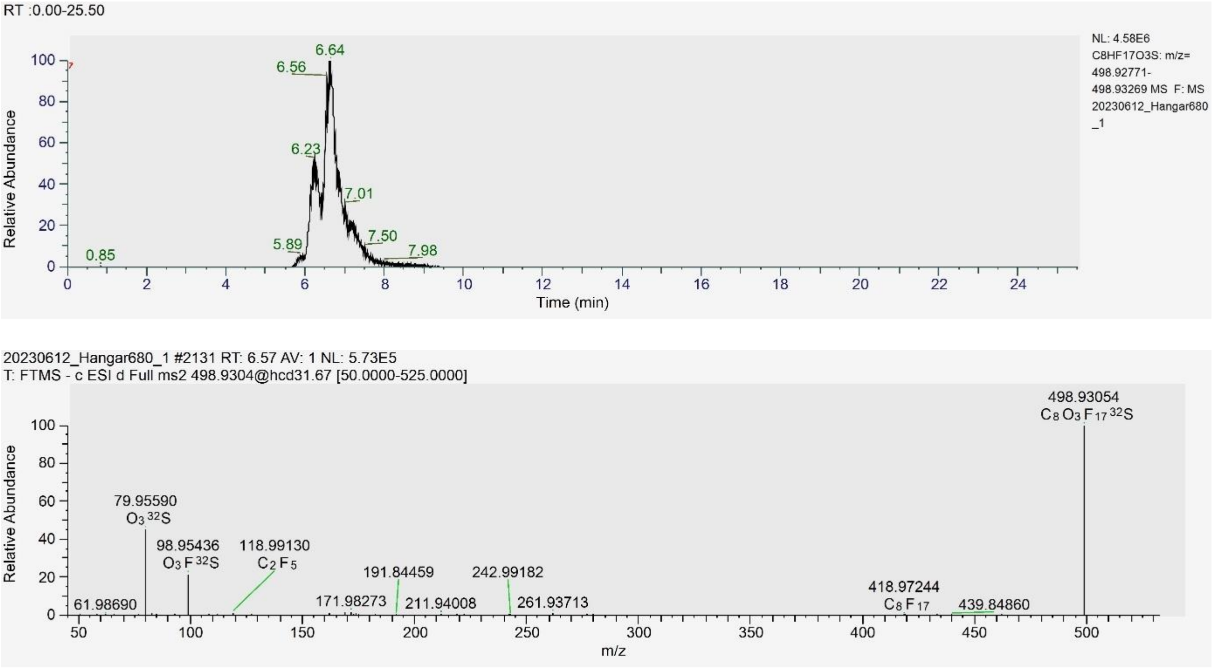

Standard

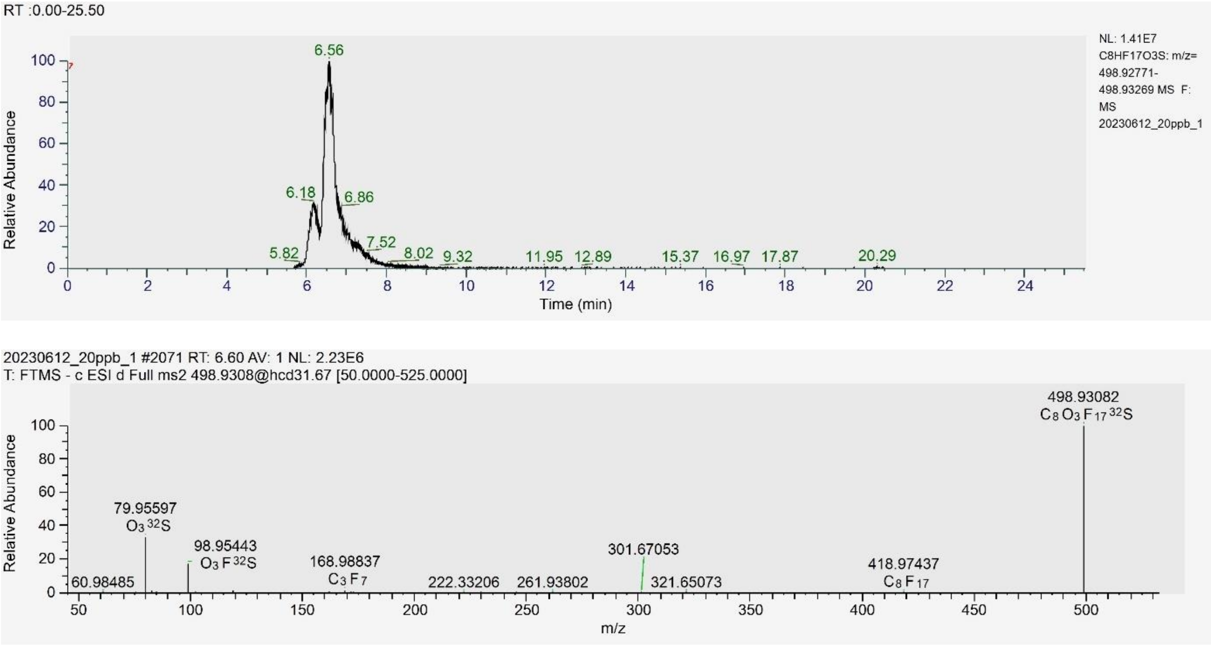

Figure S4. Confirmation of **PFOS** identification in Sample 5 (top) by comparison with reference standard (bottom). Retention times match (0.08 min) as well as the dominant fragments in the spectrum.

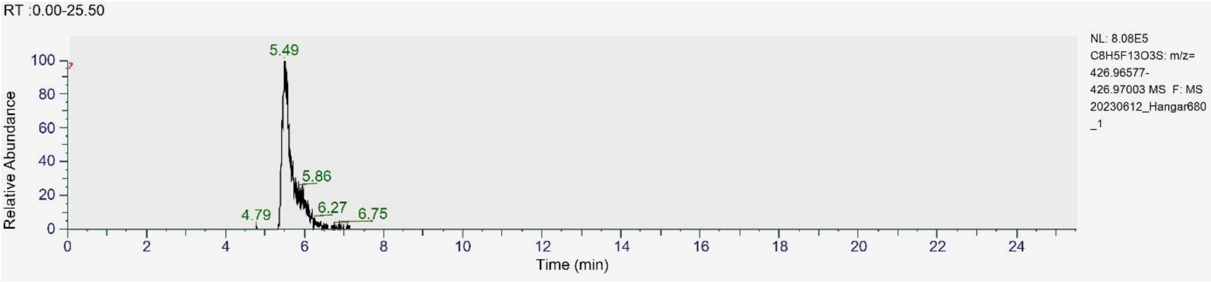

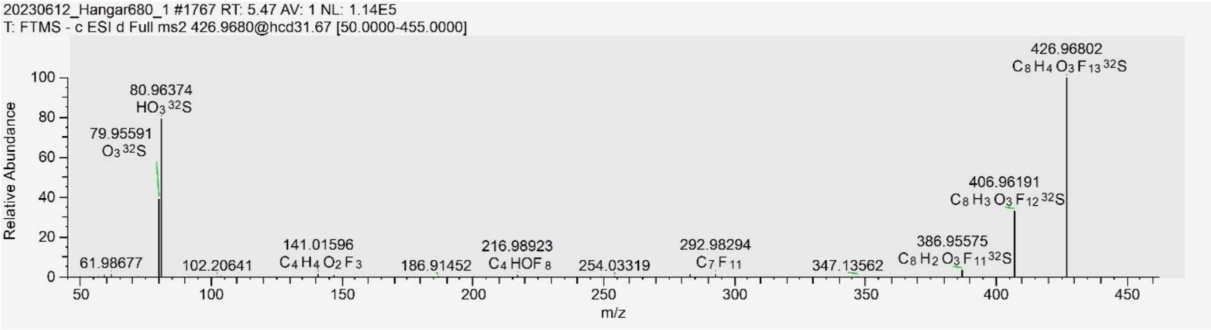

Standard

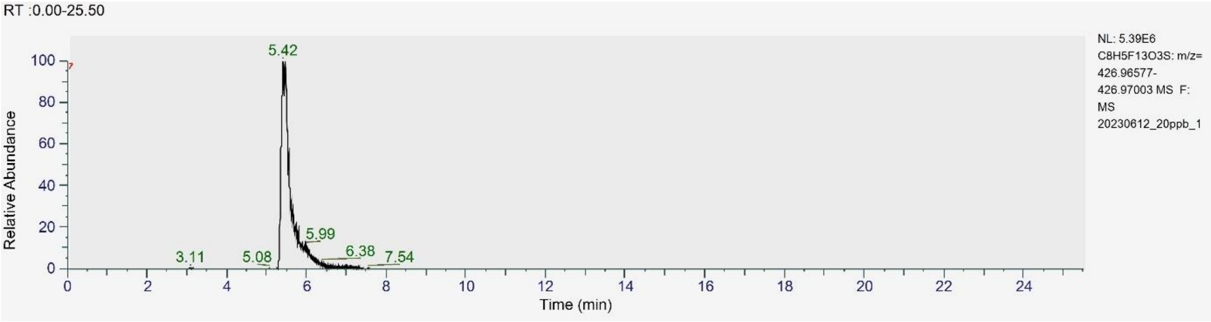

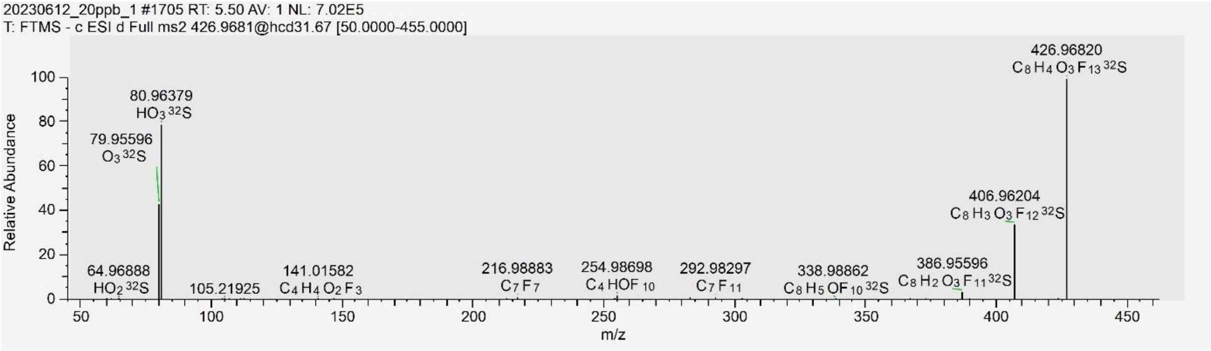

Figure S5. Confirmation of **6:2 FTS** identification in Sample 5 (top) by comparison with reference standard (bottom). Retention times match (0.07 min) as well as the dominant fragments in the spectrum.

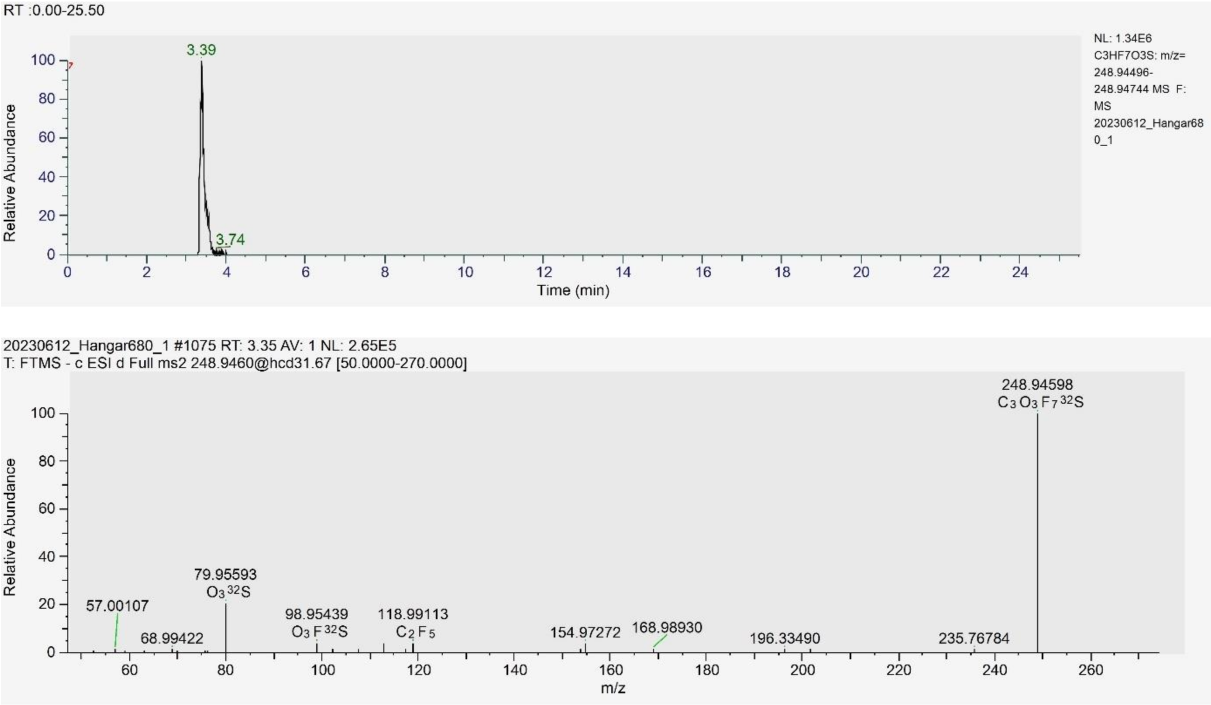

Figure S6. Confirmation of **PFPrS** identification in Sample 5. The dominant fragments in the spectrum match those reported in the literature.

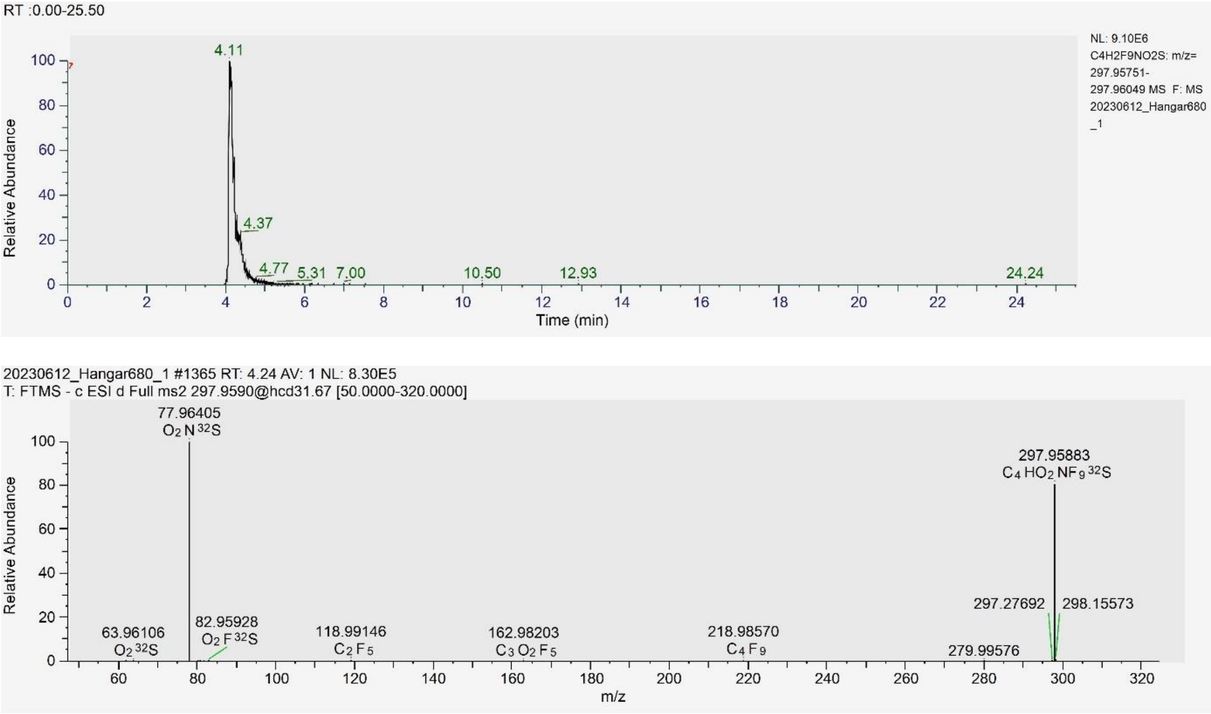

Figure S7. Confirmation of **FBSA** identification in Sample 5. The dominant fragments in the spectrum match those reported in the literature.

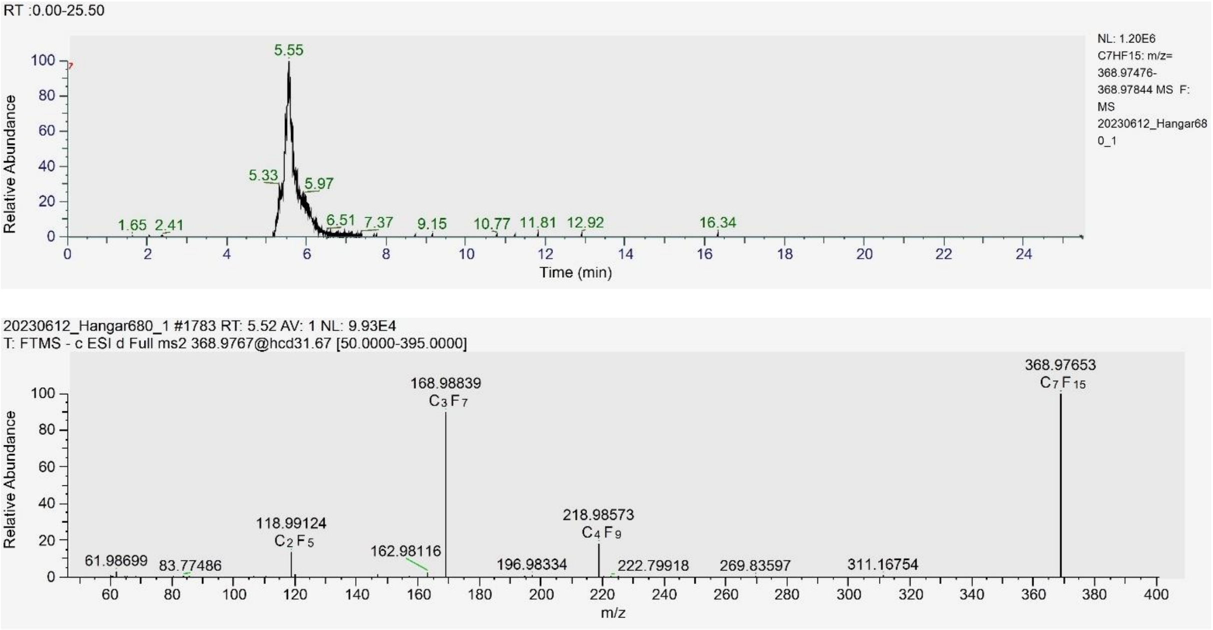

Figure S8. Confirmation of **1*H*-Perfluoroheptane** identification in Sample 5.

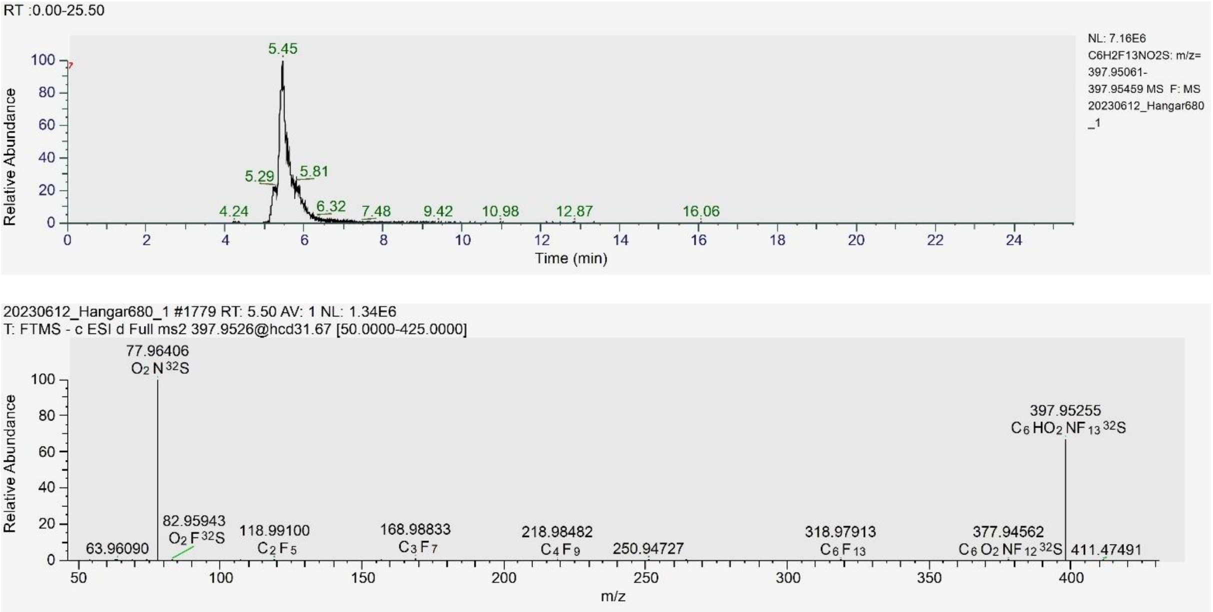

Figure S9. Confirmation of **FHxSA** identification in Sample 5. The dominant fragments in the spectrum match those reported in the literature.

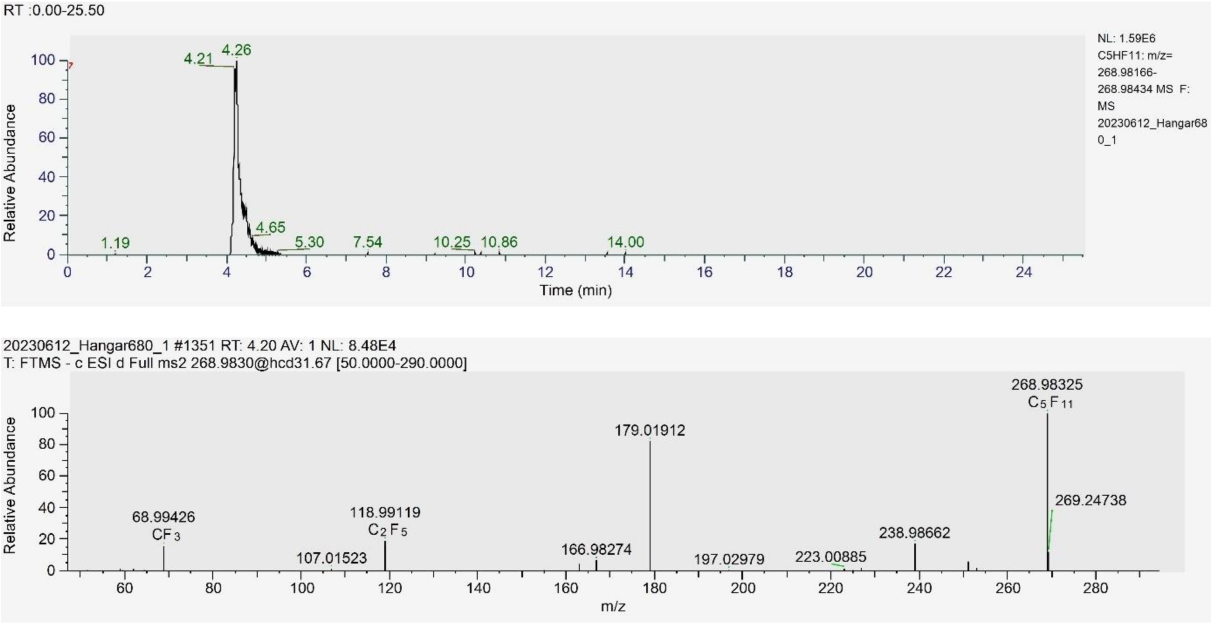

Figure S10. Confirmation of **1*H*-Perfluoropentane** in Sample 5.

Table S4. Summary of observed fragment ions for the identified PFAS compounds, including accurate masses, mass errors (ppm), and reference information to support compound identification.

Compounds	Fragments	Experimental *m/z*	Delta *m/z* (ppm)	References
PFBS	FO_3S^-<br>SO_3^-	98.9544<br>79.9560	-4.5<br>-3.2	Analytical standard
PFPeS	$C_2F_5^-$<br>FO_3S^-<br>SO_3^-	118.9912<br>98.9543<br>79.9559	-2.0<br>-3.4<br>-4.7	Analytical standard
PFHxS	$C_3F_7^-$<br>FO_3S^-<br>SO_3^-	168.9883<br>98.9544<br>79.9559	0.3<br>-3.2<br>-4.7	Analytical standard
PFOS	$C_8F_{17}^-$<br>FO_3S^-<br>SO_3^-	418.9724<br>98.9544<br>79.9560	0.3<br>-3.1<br>-4.6	Analytical standard
6:2 FTS	$C_8H_3F_{12}O_3S^-$<br>$C_8H_2F_{11}O_3S^-$<br>$C_7F_{11}^-$<br>$C_4H_4O_2F_3^-$<br>HO_3S^-<br>SO_3^-	406.9619<br>386.9558<br>292.9829<br>216.9892<br>80.9637<br>79.9559	3.2<br>3.6<br>3.6<br>-0.9<br>-4.4<br>-4.5	Analytical standard
FPrSA	$C_3F_7^-$ *<br>FO_2S^-<br>NO_2S^-	168.9884<br>82.9595<br>77.9641	1.04<br>-2.6<br>-4.6	Dewapriya *et al.* (2023)
PFPrS	$C_2F_5^-$ *<br>FO_3S^-<br>O_3S^-	118.9911<br>98.9544<br>79.9559	-2.9<br>-2.8<br>-4.2	Wu *et al.* (2022)
FBSA	$C_4F_9^-$ *<br>$C_2F_5^-$ *<br>NO_2S^-	218.9857<br>118.9915<br>77.9641	2.8<br>-0.1<br>-4.8	mzCloud [3]
1*H*-Perfluoroheptane	$C_4F_9^-$ *<br>$C_3F_7^-$ *<br>$C_2F_5^-$ *	218.9857<br>168.9884<br>118.9912	3.0<br>0.7<br>-1.9	Logical fragmentation pathways were evaluated using ChemDraw
FHxSA	$C_6F_{13}^-$ *<br>$C_4F_9^-$ *<br>NO_2S^-	318.9791<br>218.9848<br>77.9641	1.4<br>-1.2<br>-4.7	Dewapriya *et al.* (2023)
1*H*-Perfluoropentane	$C_2F_5^-$ *<br>CF_3^- *	118.9912<br>68.9943	-2.4<br>-5.9	Logical fragmentation pathways were evaluated using ChemDraw

*Highlighted: confirm that the structures are linear instead of breached, which allows the Level 2 attribution.

REFERENCES

(1) Dewapriya, P.; Nilsson, S.; Ghorbani Gorji, S.; O'Brien, J. W.; Bräunig, J.; Gómez Ramos, M. J.; Donaldson, E.; Samanipour, S.; Martin, J. W.; Mueller, J. F.; Kaserzon, S. L.; Thomas, K. V. Novel Per- and Polyfluoroalkyl Substances Discovered in Cattle Exposed to AFFF-Impacted Groundwater. *Environ. Sci. Technol.* **2023**, *57* (36), 13635–13645. https://doi.org/10.1021/acs.est.3c03852.

(2) Wu, C.; Wang, Q.; Chen, H.; Li, M. Rapid Quantitative Analysis and Suspect Screening of Per-and Polyfluorinated Alkyl Substances (PFASs) in Aqueous Film-Forming Foams (AFFFs) and Municipal Wastewater Samples by Nano-ESI-HRMS. *Water Res.* **2022**, *219*. https://doi.org/10.1016/j.watres.2022.118542.

(3) Thermo Fisher Scientific. *mz Cloud*. https://www.mzcloud.org/ (accessed 2025-10-26).
